# Physicochemical and nutritional properties of rice as affected by parboiling steaming time at atmospheric pressure and variety

**DOI:** 10.1002/fsn3.600

**Published:** 2018-02-20

**Authors:** Elvire V. Zohoun, Erasmus N. Tang, Mohamed M. Soumanou, John Manful, Noel H. Akissoe, Jude Bigoga, Koichi Futakuchi, Sali A. Ndindeng

**Affiliations:** ^1^ Unité de Recherche en Génie Enzymatique et Alimentaire Laboratoire d'Etude et de Recherche en Chimie Appliquée Ecole Polytechnique d'Abomey‐Calavi Cotonou Bénin; ^2^ Faculty of Science University of Yaoundé‐I Yaoundé Cameroon; ^3^ Africa Rice Center Bouake Côte d'Ivoire; ^4^ Faculté des Sciences Agronomiques Ecole de Nutrition et Sciences Alimentaires Cotonou Bénin

**Keywords:** gelatinization, paddy, quality, vapor exposure time, varieties

## Abstract

To elucidate the effect of different parboiling steaming time on the physicochemical and nutritional quality of rice, four varieties, NERICA1, NERICA7, IR841, and WITA4, were soaked at the same initial temperature (85°C) and steamed for 5, 15, 25, 35, and 45 min. NERICA7 steamed for 25 min recorded the highest head rice yield (71.9%). Nonparboiled IR841 recorded the shortest cooking time (17.0 min), while NERICA1 steamed for 35 min recorded the longest cooking time (26.1 min). NERICA1 steamed for 45 min was the hardest (63.2 N), while nonparboiled IR841 was the softest (28.7 N). NERICA7 recorded higher peak and final viscosities across all steaming times compared to the other varieties. NERICA7 steamed for 35 and 45 min recorded the lowest total starch (77.3%) and the highest protein (13.2%) content, respectively. NERICA7 steamed for 25 and 45 min recorded the highest phosphorus (0.166%), magnesium (572 mg/kg), and potassium (2290 mg/kg) content, respectively. We conclude that, depending on desired physicochemical and nutritional properties, specific varieties and steaming times can be selected to achieve those outcomes.

## 1 INTRODUCTION

Rice is grown for both subsistent and cash in most parts of the world. In Africa, the sativas and NERICAs (New Rice for Africa) are some of the most cultivated varieties. The sativas are cultivated mainly in irrigated/lowland production systems. NERICA is an interspecific variety, derived from *Oryza glaberrima* steud (Africa rice species) and *Oryza sativa* species (Asian rice species). The NERICAs were developed for both irrigated/lowland and upland production systems. High‐yielding rice varieties that are adapted to upland rice production systems are more likely to have a greater impact in sub‐Sahara Africa (SSA) because this production system can be accessed by a larger part of the population and does not require high investment to develop compared to irrigated/lowland production systems. However, upland production systems also have their own challenges especially as changing climatic conditions may impact on yield and grain quality (Mapiemfu et al., 2017; Tanaka et al., 2017). The role of rice in reversing food insecurity and enhancing household livelihoods in SSA was recognized during the 2008 food crises (Seck, Tollens, Wopereis, Diagne, & Bamba, 2010). From then onwards, African governments and international institutions are making tremendous efforts toward increasing the amount of rice available to consumers through importation (Seck et al., 2010) and increasing farm and postharvest yields (Food and Agricultural Organization, 2014). Several rice varieties have been developed for different rice production systems. In addition, good practices (agronomic and postharvest) including mechanization options are also being developed or adapted to increase the quantity and quality of locally produced rice (Amponsah, Addo, Dzisi, Moreira, & Ndindeng, 2017; Ndindeng et al., 2015; Saito et al., 2013). It is worth noting that most technologies have not reached most end‐users to produce the desired impact. For this reason, milled rice in sub‐Sahara Africa (SSA) markets is of variable quality (high percentage of broken fractions, impurities, and chalky grains) and mostly sold unbranded (Demont, 2013; Demont et al., 2012).

Rice quality depends on the variety, preharvest and postharvest production, and processing methods. Consumer preference and willingness to pay for rice depends on the appearance, organoleptic quality (Akoa‐Etoa et al., 2016; Demont et al., 2012), and presumed nutritional quality. Generally, rice without impurities, translucent, with a high percentage of head rice is preferred (Akoa‐Etoa et al., 2016; Demont, 2013; Diako, Sakyi‐Dawson, Bediako‐Amoa, Saalia, & Manful, 2010). In addition, consumers are looking for rice known to have; higher amounts of nutrients, shorter cooking time, high volume expansion ratio, slender in shape, and “medium to soft” texture with a natural “popcorn” aroma after cooking (Demont et al., 2017; Tang et al., under review). To meet these preferred consumer attributes, harvested paddy is either straight milled to white milled rice or parboiled before milling. Production of white milled rice is challenging due to changing climatic conditions and poor farmer‐miller practices (Ndindeng et al., 2014). Numerous factors including the presence of fissures, chalkiness, immature grains, and mixture of varieties are responsible for breakage during milling (Bergman, Bhattacharya, & Ohtsubo, 2004; Buggenhout, Brijs, Celus, & Delcour, 2013; Counce et al., 2005). However, in the recent years, production and processing gaps have been identified and the production of good quality rice is increasing in the subregion especially in areas where improved technologies have been deployed and adopted (Zossou, Mele, Vodouhe, & Wanvoeke, 2009; Fofana et al., 2011; Futakuchi, Manful, & Takeshi, 2013; Ndindeng et al., 2015; Africa Rice Center (AfricaRice), 2015). Improved rice parboiling techniques are now being disseminated in Benin, Côte d'Ivoire, and Nigeria for enhancing the physicochemical and nutritional quality of locally produced rice (Africa Rice Center (AfricaRice), 2015, 2016). Parboiling at atmospheric pressure is the hydrothermal treatment of paddy by soaking in hot water (a few degrees below its gelatinization temperature for 16 hr), steaming the hydrated paddy for 25 min followed by drying to 14% moisture content before dehusking and polishing (Graham‐Acquaah, Manful, Ndindeng, & Tchatcha, 2015; Ndindeng et al., 2015). Due to parboiling, the physical, chemical, textural, pasting, cooking, and nutritional properties of the milled rice are bound to change. Physical changes include increased head rice yield (Buggenhout et al., 2013), grain translucency, and decreased chalkiness due to starch pregelatinization (Bhattacharya, 1969, 2004; Delcour & Hoseney, 2010; Lamberts, Gormand, Deryck, & Delcour, 2009; Patindol, Newton, & Wang, 2008), increased grain hardness, and reduced grain breakage due to swelling of the starchy endosperm during gelatinization, which heals the pre‐existing defects (Ndindeng et al., 2015; Newton, Wang, & Mauromoustakos, 2011). Chemical changes include lower glycemic index, high‐resistant starch content, and high contents of B vitamins (Jenkins, Wolever, & Jenkins, 1988; Manful, Grimm, Gayin, & Coker, 2008; Newton et al., 2011; Odenigbo, Ndindeng, Nwankpa, Woin, & Ngadi, 2013; Zohoun et al., 2018). In addition, parboiling leads to development of some unique aromatic and textural characteristics that are appealing to certain groups of consumers (Demont et al., 2012; Heinemann, Behrens, & Lanfer‐Marquez, 2006; Prom‐U‐Thai, Rerkasem, Cakmak, & Huang, 2010). Some authors have reported on the optimization of soaking temperature and steaming conditions for parboiling, but the response variables were mostly related to the physical and textural properties of the rice (Graham‐Acquaah et al., 2015; Himmelsbach, Manful, & Coker, 2008; Ndindeng et al., 2015). Little information exists on the effect of varying parboiling steaming time under atmospheric pressure on the physicochemical and nutritional properties of milled parboiled rice although this information may be critical in understanding the steaming time that is rewarding for desired physicochemical and nutritional properties. It is worth noting that parboiling under atmospheric pressure is widely practiced in West Africa. Manful et al. (2008) reported on changes in thiamine and riboflavin content in parboiled rice. However, the maximum steaming time used for that study was 12 min which was lower than what is used by rice processors in the region (Ndindeng et al., 2015). As it has been demonstrated that, during parboiling, the water‐soluble nutrients diffuse from the husk and outer layer of the rice grain into the endosperm with some authors proposing parboiling as a method of rice fortification (Fukai, Godwin, Rerkasem, & Huang, 2008; Rerkasem, Cakmak, & Huang, 2010), it is important to demonstrate how increasing steaming time affects physicochemical and nutritional properties of different rice varieties. The identification of rice varieties with special properties such as increased protein and mineral contents may be interesting for researchers wishing to use parboiling as a method of rice fortification and the production of rice for people with special needs such as malnourished children or diabetics.

## 2 MATERIALS AND METHODS

### 2.1 Rice varieties

Two upland NERICA rice varieties (1 and 7), developed and disseminated by Africa Rice Center (AfricaRice) in Africa, together with two lowland sativa varieties (IR841 and WITA4) were obtained from the Genetic Resource Unit at AfricaRice. These rice varieties were selected because they are widely cultivated in Benin, recorded very low head rice yield when straight milled (18.8%), and belonged to two distinct amylose classes. NERICA7 and IR841 were from the intermediate amylose class (20–25%), while NERICA1 and WITA4 were from the high amylose class (>25%). The rice varieties were planted in their respective production systems at AfricaRice experimental plots in Cotonou, Benin, in May and harvested in August 2014 under good agronomic practices while maintaining the same recommended levels of inorganic fertilizer application rate and frequency for both production systems (Tanaka, Diagne, & Saito, 2015). Harvesting was carried out when the grain moisture was 20%–22% and dried to a moisture of 14% before used.

### 2.2 Parboiling

Parboiling was performed as previously reported (Graham‐Acquaah et al., 2015; Ndindeng et al., 2015) with slight modifications. Briefly, Paddy was cleaned, weighed, washed, and transferred to soaking pots of 5‐L capacity made from stainless steel. The paddy (1.2 kg) was heated in 3L of deionized water on a hot plate (IKA^®^ C‐MAG HS 10) set at 400°C to obtain an initial soaking temperature of 85°C. At 85°C, the pot was removed from the hot plate and left to cool to ambient temperature (±26°C) during a period of 16 hr. The soaked paddy was drained, divided into five equal portions, and steamed for the following preset steaming times: 5, 15, 25, 35, and 45 min under atmospheric pressure. Steaming was performed in the perforated basket suspended at approximately 10 cm from the boiling water level in a steaming pot. The basket was left open until steam started to emanate through the grains, at which point the lid was closed and timing started. Steaming was terminated at the preset steaming time by immediately removing the paddy from the steaming pot. All steaming experiments were replicated twice.

### 2.3 Drying

Steamed paddy was evenly sun‐dried on labeled wooden plates and mixed every 30 min while progressively monitoring the moisture content of the grains in a single kernel moisture tester (Kett model, PQ‐510). Sun drying was halted at between 16 and 18%, and drying continued in the shade to final moisture content of 14%.

### 2.4 Milling

Duplicates of nonparboiled (neither soaked nor steamed) and parboiled paddy were weighed and hulled in a THU‐34A Satake testing rice husker (Satake, Hiroshima, Japan). Brown rice was polished in a Recipal 32 rice whitener (Yamamoto Co., Higashine, Japan), and the milled rice obtained was weighed and graded into whole‐ and broken‐rice fractions in a rotating cylinder test rice grader (Satake, Hiroshima, Japan). The weight of the whole grains was recorded, and all samples sealed in well‐labeled envelopes and kept at ambient temperature prior to quality analysis.

### 2.5 Preparation of rice flour

For each sample, 5 g of grains were ground to fine powder in a grinder (UDY cyclone mill; Fort Collins, CO, USA) fitted with a fine sieve of 0.5 mm mesh size. The prepared rice flour was reserved for rheological, chemical, and nutritional analyses.

### 2.6 Physicochemical analysis

#### 2.6.1 Milling recoveries

Head rice yield (HRY) was computed in duplicates for each cultivar as shown in equation 1 below:

(1) $$\mathrm{HRY}\left(\%\right)=\frac{\left(\text{Weight of whole grains}\right)}{\left(\text{Weight of paddy}\right)}\times 100.$$

#### 2.6.2 Chalkiness

Percent chalky grains was determined on a 50‐g sample using S21 rice statistical analyzer (LKL Technologia, Santa Cruz do Rio Pardo, Brazil), calibrated with the reference sample (Tinto) supplied by the manufacturer as previously reported (Graham‐Acquaah et al., 2015; Ndindeng et al., 2015).

#### 2.6.3 Pasting properties

The pasting characteristics of the prepared rice flour were determined using a Rapid Visco Analyzer; model super4 (RVA, Newport Scientific, Warriewood NSW, Australia). The Rapid Visco Analyzer (RVA) was switched on and allowed to cool to 8**°**C before the start of the analysis. The rest of the test performed in duplicates as previously reported (Graham‐Acquaah et al., 2015; Ndindeng et al., 2015). Pasting parameters determined from the RVA curve included the following: peak viscosity (PV), trough (TV), and final viscosities (FV). Breakdown viscosity (BV) was calculated as the difference between PV and TV, while setback viscosity (SBV) was the difference between the FV and PV.

#### 2.6.4 Texture

Texture profile analysis (TPA) was performed as previously reported (Graham‐Acquaah et al., 2015; Ndindeng et al., 2015) with a texture analyzer (TA‐XT‐Plus, Stable Micro Systems Ltd., Surrey, UK) whose cylindrical probe was 35 cm in diameter. Grains were cooked for 20 min, and immediately after cooking, three grains were arranged in parallel on the center of the sample platform grid for analysis during which a two‐cycle compression force versus time was used to compress the grains to 90% deformation at a pretest speed of 0.5 mm/s and a post‐test speed of 10 mm/s. The texture parameters recorded from the generated TPA curve were hardness and stickiness.

#### 2.6.5 Apparent amylose content (AAC)

Apparent amylose content (AAC) was determined using an AutoAnalyzer 3 (Seal Analytical, Norderstedt, Germany) as previously reported (Ndindeng et al., 2015) following the standard iodine colorimetric method ISO 6647‐2‐2011 (ISO, 2011). Duplicate 100 mg of prepared flour of each rice sample was weighed into a 100‐ml volumetric flask, and standards of well‐known amylose contents (IR65, IR64, IR8, IR24, and IR24/65 from IRRI (Los Baños, Philippines), and 465, 466, and 467 supplied by the Institute for Reference Material and Measurement (Geel, Belgium)) also measured in the same way for the construction of the standard curve. Flour (100 mg) of each sample was mixed in 1 ml of 99% ethanol plus 9 ml of 1 mM NaOH, and then the starch gelatinized by heating for exactly 10 min. After cooling, the contents were completed to 100 ml with deionized water and 8 ml from each flask was used for analysis. Optical densities were read at 600 nm.

#### 2.6.6 Alkaline spreading value

The alkaline spreading value (ASV) was determined using the method of Little, Hilder, & Dawson, 1958 with slight modifications. Briefly, six grains of milled rice were immersed in 10 ml of 1.7% KOH in a transparent Petri dish and incubated at room temperature for 23 hr. The degree of grain dispersion was observed and scored as detailed in Jennings, Coffman, & Kauffman, 1979. Based on the ASV score, samples were assigned to existing gelatinization temperature (GT) classes. Samples with ASV score of <4 had high GT (75–79), those with ASV of <6 had intermediate GT (70–74), and those with ASV of >6 had low GT (55–69).

#### 2.6.7 Cooking time

Cooking time was carried out as previously reported (Fofana et al., 2011). Briefly, duplicate 5 g of whole grains was cooked in deionized water for 10 min at 400**°**C. From the 11th min, 10 grains were removed every 1 min and gently pressed between two glass Petri dishes to observe for opaqueness. The presence of opaque centers indicated uncooked grains, while transparent centers signified cooked grains. The time when all the 10 grains had transparent centers corresponded to the cooking time.

#### 2.6.8 Water uptake ratio (WUR) and volume expansion ratio (VER)

Eight grams of uncooked head rice was weighed in duplicates into a 50‐ml measuring cylinder, and the height (H1) and weight (W1) were measured. The rice was then cooked in a cooking mesh basket (inside 400‐ml beaker in excess water) on a hot plate set at medium heat level for the determined cooking time, and the height (H2) and weight (W2) of the cooked grains were taken with the same cylinder. Calculations were as follows:

(2) $$\text{Water uptake ratio (WUR)}=\frac{W2-W1}{8\;g}$$

(3) $$\text{Volume expansion ratio (VER)}=\frac{H2}{H1}.$$

### 2.7 Total starch, lipid, protein, and mineral analysis

Total starch determination proceeded as described in the Megazyme total starch assay kit (K‐TSTA, Megazyme Int. Co. Wicklow, Ireland) based on AOAC (1984) method 996.11 and AACC method 76‐13.01. The lipid content was determined by the Soxhlet method (AOAC, 1984) using petroleum ether as the extraction solvent. Total nitrogen was determined by the Kjeldahl method using 6.25 as the total protein nitrogen conversion factor. Mineral content (P, K, Ca, Mg, Na, and Fe) was determined by atomic absorption spectrophotometer (Varian Vista, Victoria, Australia). All samples were analyzed in triplicates.

### 2.8 Statistical analysis

Data collected were entered into an Excel spreadsheet (Office 365, Microsoft Corporation). Graphs of viscosity/operating temperature against time plots were generated for all treatment. The effect of variety, steaming time, and interaction of variety and steaming time on head rice yield (HRY), chalkiness, alkaline spreading value (ASV), cooking time (CT), volume expansion ratio (VER), water uptake ratio (WUR), lipid content, apparent amylose content (AAC), cooked grain hardness, viscosity profile, protein, phosphorus, potassium, calcium, magnesium, and sodium contents were investigated using multivariate regression analysis. Means of treatments were compared using Fisher's least significant difference multiple comparison test. The least square (LS) means following ranking are reported. Pearson correlations were determined between studied parameters. The statistical program used for the analysis was XLSTAT^™^ Premium software for Windows^®^ version 19.5 (2017) (Addinsoft SARL, 2017 Paris, France). All analyses were carried out at the 5% significance level.

## 3 RESULTS AND DISCUSSION

In order to elucidate the effect of different parboiling steaming time on the physicochemical and nutritional quality of rice, four varieties, NERICA1, NERICA7, IR841, and WITA4, were soaked at the same initial temperature (85°C) and steamed for 5, 15, 25, 35, and 45 min. Except for calcium content, all studied parameters [head rice yield (HRY), chalkiness, alkaline spreading value (ASV), cooking time (CT), volume expansion ratio (VER), water uptake ratio (WUR), lipid content, apparent amylose content (AAC), cooked grain hardness, viscosity profile, protein, phosphorus, potassium, calcium, magnesium, and sodium contents] were affected by variety or parboiling steaming time or variety and parboiling steaming time (*p* < .05). Among the studied varieties and based on physicochemical and nutritional properties studied, NERICA7 was ranked first followed by NERICA1, WITA4, and IR841 in decreasing order (Table 1). Likewise, 25‐min steaming time was ranked first followed by 5, 15, 0, 35, and 45 min (Table 2).

**Table 1:** Effect of variety on the physicochemical and nutritional properties of some rice varieties cultivated in Benin parboiled at atmospheric pressure

Variety	Head rice yield (%)	Chalkiness (%)	Final viscosity (cP)	Hardness (N)	Stickiness (gsec)	Apparent Amylose (%)
NERICA7	59.7 a^a^	6.4 b	2,080 a	46.5b	−6.8 a	21.3 d
NERICA1	50.7 b	9.8 a	770 c	55.8 a	−0.9 c	28.5 a
WITA4	49.9 b	4.1 c	1,231 b	41.6 c	−2.7 b	26.4 b
IR841	46.3 b	3.2 c	1,190 b	42.5 c	−2.3 b	23.4 c
Pr > *F* (Model)	<0.0001	<0.0001	<0.0001	<0.0001	<0.0001	<0.0001
Adjusted *R* ^2^	.86	.98	.98	.85	.76	.83

	Alkaline spreading value	Cooking time (min)	Volume expansion ratio	Water uptake ratio	Total starch (%)	Lipid (%)
NERICA7	6.1 c	23.3 a	2.2 c	1.1 b	80.0 b	0.93 c
NERICA1	5.8 d	23.7 a	2.3 a	1.1 b	88.7 a	0.73 d
WITA4	6.9 a	18.7 b	2.4 b	1.4 a	82.2 b	1.60 a
IR841	6.6 b	18.2 b	1.4 d	1.4 a	88.1 a	1.30 b
Pr > *F* (Model)	<0.0001	<0.0001	<0.0001	<0.0001	0.0001	<0.0001
Adjusted *R* ^2^	.73	.85	.88	.62	.42	.73

	Protein (%)	Phosphorus (%)	Potassium (mg/kg)	Calcium (mg/kg)	Magnesium (mg/kg)	Sodium (mg/kg)
NERICA7	12.8 a	0.146 a	2,031 a	94.1 a	502 a	67.6 b
NERICA1	11.0 b	0.136 a	1,847 ab	90.0 a	432 b	83.0 a
WITA4	9.20 c	0.110 c	1,541 c	93.6 a	344 c	45.0 c
IR841	8.45 d	0.122 b	1,800 b	89.0 a	361 c	49.7 c
Pr > *F* (Model)	<0.0001	<0.0001	<0.0001	0.459	<0.0001	0.001
Adjusted *R* ^2^	.92	.57	.56	.007	.58	.37

a Indicates that least square means with the same letter are significantly different at the 5% level.

**Table 2:** Effect of atmospheric pressure parboiling steaming time on the physicochemical and nutritional properties of four rice varieties cultivated in Benin

Steaming time	Head rice yield (%)	Chalkiness (%)	Final viscosity (cP)	Hardness (N)	Stickiness (gsec)	Apparent amylose (%)
25	64.2 a^a^	0.00 b	755 d	51.2 a	−2.4 c	25.2 ab
5	32.7 b	0.39 b	2,095 b	45.6 b	−3.8 b	25.0 ab
15	61.3 a	0.01 b	1,141 c	50.0 a	−3.0 bc	25.1 ab
45	67.3 a	0.00 b	389 f	50.8 a	−2.3 c	24.2 b
0	18.8 c	34.9 a	2,985 a	33.4 c	−5.2 a	25.5 a
35	65.7 a	0.00 b	542 e	48.6 a	−2.4 c	24.5 ab
Pr > *F* (Model)	<0.0001	<0.0001	<0.0001	<0.0001	<0.0001	<0.0001
Adjusted *R* ^2^	.86	.98	.98	.85	.76	.83

	Alkaline spreading value	Cooking time (min)	Volume expansion ratio	Water uptake ratio	Total starch (%)	Lipid (%)
25	6.4 a	21.1 b	2.03 bc	1.2 b	84.5 a	1.13 a
5	6.5 a	20.5 bc	2.09 bc	1.2 b	85.0 a	1.14 a
15	6.5 a	20.9 b	2.08 bc	1.2 b	85.7 a	1.26 a
45	6.5 a	21.0 b	2.02 c	1.1 b	85.5 a	1.25 a
0	5.5 b	19.8 c	2.28 a	1.5 a	84.7 a	0.73 b
35	6.5 a	22.5 a	2.17ab	1.2 b	83.2 a	1.31 a
Pr > *F* (Model)	<0.0001	<0.0001	<0.0001	<0.0001	0.000	<0.0001
Adjusted *R* ^2^	.73	.85	.88	.62	.42	.73

	Protein (%)	Phosphorus (%)	Potassium (mg/kg)	Calcium (mg/kg)	Magnesium (mg/kg)	Sodium (mg/kg)
25	10.5 a	0.134 a	1,961 a	94.0 a	462 a	62.9 ab
5	10.6 a	0.133 a	1,923 a	88.0 a	428 a	75.0 a
15	10.4 a	0.128 a	1,913 a	92.2 a	428 a	59.9 ab
45	10.5 a	0.140 a	1,981 a	90.3 a	444 a	56.1 b
0	9.90 b	0.102 b	1,198 b	98.6 a	284 b	62.7 ab
35	10.3 a	0.133 a	1,852 a	86.8 a	412 a	51.3 b
Pr > *F* (Model)	<0.0001	<0.0001	<0.0001	0.459	<0.0001	0.001
Adjusted *R* ^2^	.92	.57	.56	.007	.58	.37

a Indicates that least square means with the same letter are significantly different at the 5% level.

### 3.1 Head rice yield (HRY)

NERICA7 steamed for 25 min recorded the highest HRY (71.9%), while nonparboiled WITA4 recorded the lowest (7.1%) (Table 1). The highest HRY for IR841, WITA4, NERICA1, and NERICA7 was recorded after 25, 25, 45, and 25 min of steaming, respectively. This finding indicates that 25‐min steaming time could be used for all studied varieties except NERICA1 when a soaking temperature of 85°C is used. Optimum steaming time of 25 min has been proposed for TOX3145 (Ndindeng et al., 2015). Graham‐Acquaah et al. (2015) indicated that irrespective of steaming time, if initial soaking temperature did not exceed 50___C, HRY for NERICA1 variety was always below that recorded for the nonparboiled counterpart. Parboiling conditions thus depend on rice variety and go to confirm the concept that parboiling conditions are related to gelatinization temperature, which is strictly variety‐specific (Marshall, 1993). In this study, a weak but significant positive correlation was recorded between HRY and ASV (*R* = .25; *p* = .03) suggesting that samples with low GT tended to produce higher HRY (Table 4). Rice with higher HRY attracts better market price than rice with high broken‐rice yield (Khush, Paule, & De Cruz La, 1979; Koutroubas, Mazzini, Pons, & Ntanos, 2004; Tang et al., under review) indicating higher preference by most consumers especially in urban markets (Akoa‐Etoa et al., 2016; Demont et al., 2012). Strategies to increase HRY continue to gain attention in rice industries around the world especially now that rice breakage seems to be increasing due to changing climatic conditions (Ndindeng et al., 2014; Zhang, Zhang, Yang, & Zhang, 2008). The reduction in rice breakage depends on selecting the best soaking temperature and steaming time (Graham‐Acquaah et al., 2015; Sareepuang, Siriamornpun, Wiset, & Meeso, 2008), using the best parboiling equipment that allows the uniform distribution of heat during soaking and steaming (Ndindeng et al., 2015), proper drying to reduce cracks (Rao, Bal, & Goswami, 2007), and milling with a rubber roll‐type mill (Bhattacharya, 1969, 2004; Delcour & Hoseney, 2010).

### 3.2 Chalkiness

Chalkiness decreased with parboiling for all varieties as expected. In the nonparboiled form (steaming time = 0), NERICA1 recorded the highest percentage of chalky grains (58.2%) followed by NERICA7 > WITA4 > IR841 in that order (Table 3). When steamed for 5 min, chalkiness decreased by at least 92% for all the varieties and 100% after 15 min of steaming confirming earlier studies (Manful et al., 2008; Ndindeng et al., 2015). Chalkiness in the studied samples was negatively correlated with phosphorus (*R* = −3.8; *p* < .001), potassium (*R* = −.50; *p* < .0001), magnesium (*R* = −.47; *p* < .0001), hardness (*R* = −.51; *p* < .0001), and HRY (*R* = −.59; *p* < .0001) (Table 4). This suggests that varieties that can accumulate more phosphorus, potassium, and magnesium in their grains will record reduced grain chalkiness, increased hardness, and HRY. This was typical of NERICA7 used in this study. Low chalky values have been reported to indicate better sensory quality both for nonparboiled and parboiled rice (Kim, Lee, Kim, & Kim, 2000). Chalkiness in rice is controlled genetically (Khush et al., 1979; Lanning, Siebenmorgen, Counce, Ambardekar, & Mauromoustakos, 2011; Li et al., 2004), but its extent can be affected by the environmental conditions experienced during the grain‐filling period (Ndindeng et al., 2014; Yamakawa, Hirose, Kuroda, & Yamaguchi, 2007) and disease attack (Mapiemfu et al., 2017). Mapiemfu et al. (2017) indicated that chalkiness was higher in upland production systems compared to lowland and irrigated systems, and the main reason proposed for this was water stress associated with this production system explaining why the upland varieties recorded higher chalky values. Chalkiness leads to high grain breakage during milling (Perez, Juliano, Liboon, Alcantara, & Cassman, 1996; Patindol & Wang, 2002), lowers the esthetic look of rice grains, and is considered undesirable by most rice consumers, thus reducing the marketability of chalky rice (Khush et al., 1979). Parboiling is thus a good strategy to upgrade the quality rice as this eliminates chalkiness.

**Table 3:**
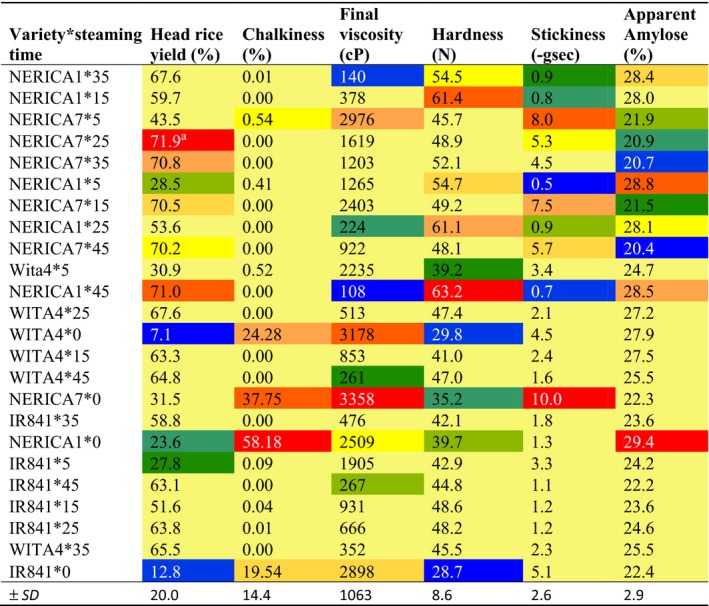
Interactive effect of variety and parboiling steaming time at atmospheric pressure on head rice yield, chalkiness, final viscosity, grain hardness, stickiness, and apparent amylose content of some varieties cultivated in Benin

Values in red are highest, while those in blue are lowest.

**Table 4:** Relationship between physicochemical, cooking, and nutritional properties of some rice varieties cultivated in Benin

Variables	HRY	Chalk	FV	Hard	Stick	AAC	ASV	CT	VER	WUR	TSC	Lipid	Protein	P	K	Ca	Mg
HRY	1.00																
Chalk	−0.59*	1.00															
FV	−0.69*	0.60*	1.00														
Hard	0.59*	−0.51*	−0.65*	1.00													
Stick	−0.17	0.23	0.68*	−0.42*	1.00												
AAC	−0.18	0.17	−0.27***	0.24***	−0.60*	1.00											
ASV	0.25***	−0.70*	−0.36***	0.00	−0.13	−0.14	1.00										
CT	0.31***	−0.09	−0.13	0.57*	0.11	0.04	−0.30***	1.00									
VER	−0.03	0.26***	0.05	0.21	−0.01	0.47*	−0.34***	0.51*	1.00								
WUR	−0.46*	0.33***	0.32***	−0.68*	0.07	0.10	0.15	−0.64*	−0.15	1.00							
TSC	−0.13	0.03	−0.26***	0.17	−0.43*	0.36***	−0.05	−0.07	−0.13	−0.07	1.00						
Lipid	0.23	−0.41*	−0.33***	−0.09	−0.17	−0.05	0.64*	−0.42*	−0.30***	0.24***	−0.20	1.00					
Protein	0.27*	−0.01	0.17	0.39**	0.43*	−0.24***	−0.34***	0.78*	0.45*	−0.60*	−0.33***	−0.42*	1.00				
P	0.38**	−0.38*	−0.20	0.39**	0.06	−0.28***	0.03	0.46*	0.01	−0.49*	−0.03	−0.33***	0.52*	1.00			
K	0.51*	−0.50*	−0.38*	0.50*	−0.09	−0.26	0.12	0.36***	−0.14	−0.58*	0.00	−0.04	0.43*	0.75*	1.00		
Ca	−0.08	0.10	0.16	−0.18	0.15	−0.03	0.06	0.02	0.00	0.13	−0.13	−0.17	0.07	0.43*	0.24***	1.00	
Mg	0.43*	−0.47*	−0.22	0.50*	0.13	−0.27***	0.13	0.56*	0.09	−0.56*	−0.12	−0.18	0.64*	0.86*	0.76*	0.38**	1.00
Na	−0.08	0.06	0.15	0.27***	−0.02	0.14	−0.34***	0.45*	0.32***	−0.46*	0.09	−0.51*	0.42*	0.33***	0.19	0.16	0.31***

HRY, head rice yield; Chalk, chalkiness; FV, final viscosity; Hard, cooked grain hardness; Stick, cooked grain stickiness; AAC, apparent amylose content; ASV, alkaline spreading value; CT, cooking time; VER, volume expansion ratio; WUR, water uptake ratio; STC, total starch content; P, phosphorus content; K, potassium content; Ca, calcium content; Mg, magnesium content; Na, sodium content.

*p < .0001;**p < .001;***p < .05.

### 3.3 Pasting profile

**Figure 1:**
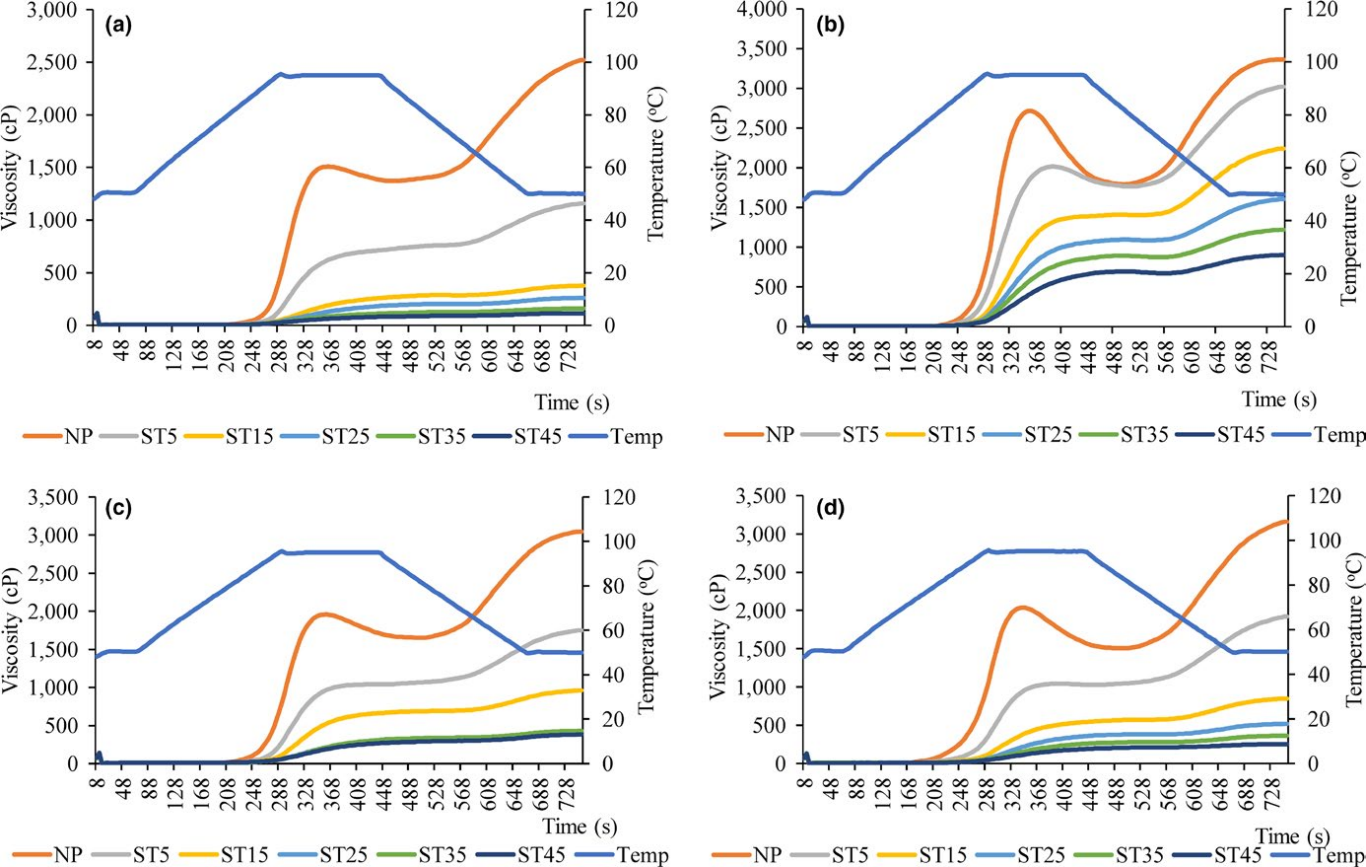
Pasting profile of (a) NERICA1, (b) NERICA7, (c) IR841, and (d) WITA4 parboiled using different parboiling steaming time at atmospheric pressure (ST5, ST15, ST25, ST25, and ST45) in comparison with nonparboiled (NP) counterparts

The pasting profiles of the flour slurries of the rice varieties studied were influenced by the steaming time and the measured viscosities (PV, FV, BV, and SBV), decreased with increasing steaming time (Figure 1). PV decreased in the order NERICA7 (b) > WITA4 (d) > IR841 (c) > NERICA1 (a), while this pattern does not change with steaming time. BV decreases in the order NERICA7 > WITA4 > IR841 > NERICA1 from 0 to 15 min and in the order NERICA1 >  WITA4 >  IR841 >  NERICA7 from 25 to 45 min. FV and SBV decreased in the order NERICA7 >  IR841 >  WITA4 >  NERICA1, and the pattern does not change with steaming time. NERICA7 demonstrated the highest PV, FV, and SBV values during the different steaming times. It also demonstrated a high BV between 0 and 15 min steaming time but the lowest BV from 25 min of steaming. These results suggest that a greater proportion of the starch granules in NERICA7 remains ungelatinized due to an unknown resistance even at severe steaming conditions, while the starch granules in the other varieties are very sensitive to heat and most get gelatinized at moderate steaming conditions. Some authors have reported on the decrease in the viscosity properties of rice flour due to parboiling (Himmelsbach et al., 2008; Newton et al., 2011; Soponronnarit, Nathakaranakule, Jirajindalert, & Taechapaorij, 2006). The decrease in viscosity parameters with increasing steaming time and as a function of rice varieties is in accord with studies by Patindol et al. (2008). The most convincing explanation for the decrease in viscosity in parboiled rice slurries points to the decrease in the number of native granules caused by partial gelatinization during the soaking and steaming operations of parboiling. In this study, final viscosity was negatively correlated with AAC (*R* = −.27, *p* < .05), TSC (*R* = −.26, *p* < .05), lipid (*R* = −.33, *p* < .05), and potassium content (*R* = −.38, *p* < .001) (Table 4).

### 3.4 Texture profile

Variety, steaming time, and the interaction between variety and steaming time influenced cooked grain hardness and stickiness for all studied varieties (*p* < .05). Overall, NERICA1 was the hardest (55.8 N), while NERICA7 was the stickiest (−6.8 gsec) (Table 1). Parboiling increased cooked grain hardness and reduced stickiness as previously reported (Biswas & Juliano, 1988; Islam, Shimizu, & Kimura, 2002; Ndindeng et al., 2015; Patindol et al., 2008). During steaming, grain hardness peaked after 25 min, while stickiness peaked after 15 min (Table 2.). NERICA1 steamed at 45 min was the hardest (63.2 N), while nonparboiled IR841 was the softest (28.7N). Nonparboiled NERICA7 was the stickiest sample (‐10 gsec), while NERICA1 steamed at 5 min was the least sticky (‐0.5 gsec) (Table 3). Cooked grain hardness was positively correlated with AAC (*R* = 22, *p* < .05), protein (*R* = .39, *p* < .001), phosphorus (*R* = .39, *p* < .001), potassium (*R* = .50, *p* < .0001), magnesium (*R* = .50, *p* < .0001), and sodium (*R* = .27, *p* < .05). Stickiness correlated negatively with AAC (*R* = −.60, *p* < .0001), total starch (*R* = −.43, *p* < .0001) and positively with protein content (*R* = .43, *p* < .0001) (Table 4). It is commonly known that high amylose rice cooks hard while low amylose rice cooks sticky. Minerals such as phosphorus, potassium, magnesium, and sodium may be involved in complex formation that stabilizes the starch structure, increases hardness, and reduces hydrolysis (Ahmadi‐Abhari et al., 2013; Singh, Dartois, & Kaur, 2010). Several authors have indicated that cooked grain hardness is due to the starch degradation ability of the hydrothermal treatment of parboiling and to the reassociation of gelatinized starch at the end of parboiling (Biswas & Juliano, 1988; Billiaderis et al. 1993; Ramesh, Ali, & Bhattacharya, 1999), and this is favored by high AAC. In addition, Derycke, Vandeputte, Vermeylen, et al. (2005) suggested that thiol amino acid or proteins with intact disulfide bonds are formed during parboiling, and this prevents the leaching of solids during cooking leading to a decrease in adhesiveness and an increase in cooked grain hardness, as stickiness was positively correlated with proteins and negatively with AAC suggesting that some proteins in the presence of low amylose will favor stickiness.

### 3.5 Apparent amylose content (AAC)

NERICA7 and IR841 were from the intermediate amylose class (20–25%), while NERICA1 and WITA4 were from the high amylose class (> 25%).Nonparboiled NERICA1 recorded the highest AAC (29.4%), while NERICA7 steamed at 45 min recorded the lowest AAC (20.4%) (Table 3). The AAC of NERICA1, WITA4, and IR841 tended to fluctuate as steaming time increased while that for NERICA7 decreased steadily. The above results indicate that AAC of parboiled samples depended on the variety and the steaming time used. The decreased AAC with increasing steaming time in this study is attributed to amylose and solid leaching during soaking and steaming (Patindol et al., 2008). The results obtained for NERICA1 confirm earlier studies that reported decreased AAC for NERICA1 for steaming times below 25 min (Graham‐Acquaah et al., 2015) and for TOX3145 steamed at 25 min (Ndindeng et al., 2015). Nonparboiled samples recorded the highest AAC (25.5%), while samples steamed at 45 min recorded the lowest (24.2%) (Table 2). Although parboiling decreased AAC, this did not affect the amylose class of the variety in the nonparboiled form except for WITA4 when it was steamed for 5 min (24.7%). Samples with lower AAC tended to record higher protein (*R* = −.24, *p* < .001), phosphorus (*R* = −.28, *p* < .001), potassium (*R* = −.26, *p* < .001), and magnesium (*R* = −.27, *p* < .001) contents (Table 4). This was mainly due to the type of variety used and the parboiling process, where there is concomitant leaching of amylose and diffusion of water‐soluble substances from the husk and pericarp into the other layers of the endosperm.

### 3.6 Cooking quality

#### 3.6.1 Alkaline spreading value (ASV)

Nonparboiled NERICA1 was of the high GT class (ASV < 4), NERICA7 and IR841 were of the intermediate GT class (ASV < 6), while WITA4 was of the low GT class (ASV >6). As expected, nonparboiled samples recorded higher GT than parboiled samples, and steaming for a longer time did not affect the GT class of the samples (Tables 2 and 5). ASV was positively correlated with lipid (*R* = .64, *p* < .0001) and negatively correlated with proteins (*R* = −.34, *p* < .001) and magnesium (*R* = −.34, *p* < .001) (Table 4). This suggests that samples with high lipid content recorded lower GT, while those with high protein content recorded higher GT. GT is the temperature at which the starch molecules begin to swell in the presence of water and heat. The above finding confirms the presence of a protein barrier linked by disulfide bonds natively present in rice or formed during parboiling or cooking that restricts heat‐induced swelling of rice starch (Derycke, Vandeputte, Vandeputte, et al., 2005).

**Table 5:**
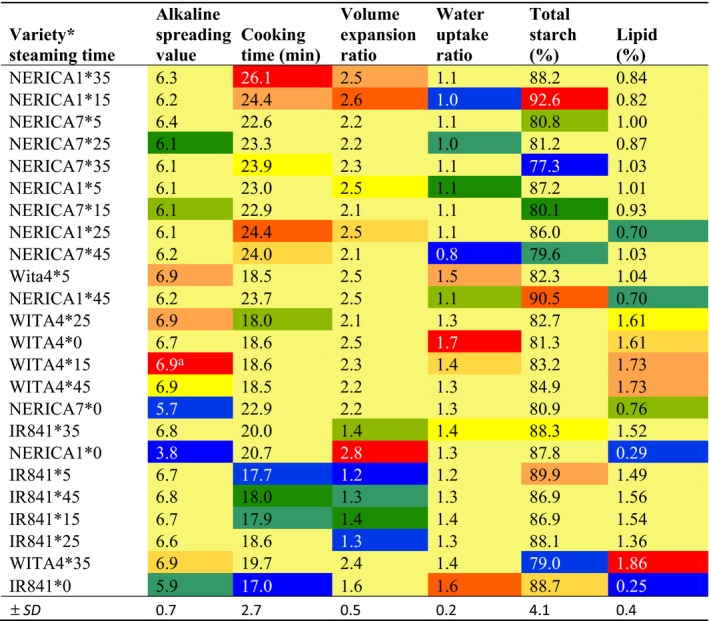
Interactive effect of variety and parboiling steaming time at atmospheric pressure on alkaline spreading value, cooking time, volume expansion and water uptake ratios, and total starch and lipid contents of some varieties cultivated in Benin

Values in red are highest, while those in blue are lowest.

#### 3.6.2 Cooking time

The time required to cook rice grains to the softness preferred for human consumption varied across varieties and steaming time (*F* = 18.79; *p* < .0001). NERICA7 and NERICA1 samples took the same time to cook (about 23.5 min), while the same could be said of WITA4 and IR841 (about 18.5 min) Samples steamed at 35 min took the longest time to cook (22.5 min), while nonparboiled samples took the shortest time (19.8 min) (Tables 1 and 2). NERICA1 steamed at 35 min recorded the longest cooking time (26.1 min), while nonparboiled IR841 recorded the shortest cooking time (17 min) (Table 5). Cooking time was positively correlated with protein (*R* = .78, *p* < .0001), phosphorus (*R* = .46, *p* < .0001), potassium (*R* = .36, *p* < .001), magnesium (*R* = .56, *p* < .0001), and sodium (*R* = .45, *p* < .0001) and negatively correlated with lipid content (*R* = −.42, *p* < .0001) (Table 4). As the cooking process goes through gelatinization and the fact that GT is a pointer to cooking time, the observations above are consistent with the presence of a protein barrier indicated in 3.6.1 above. Such a barrier (amylose or protein) may be reinforced by metal cations and phosphorylation.

#### 3.6.3 Water uptake ratio (WUR) and volume expansion ratio (VER)

Water uptake ratio (WUR) was lower for NERICA1 and NERICA7 (1.1) compared with WITA4 and IR841 (1.4). Nonparboiled samples recorded higher WUR (1.5) than parboiled samples, but increasing steaming time did not affect WUR although the lowest WUR was recorded with samples steamed at 45 min (1.1) (Tables 1 and 2). Nonparboiled WITA4 recorded the highest WUR (1.7), while NERICA7 steamed at 45 min recorded the lowest (0.8) (Table 5). WUR was negatively correlated with protein (*R* = −.60, *p* < .0001), phosphorus (*R* = −.49, *p* < .0001), potassium (*R* = −.58, *p* < .0001), magnesium (*R* = −.56, *p* < .0001), and sodium (*R* = −.46, *p* < .0001) and positively correlated with lipid (*R* = .24, *p* < .001) contents (Table 4).

Volume expansion ratio (VER) was influenced by variety, steaming time, and a combination of variety and steaming time (*F* = 24.29; *p* < .0001). Overall, NERICA1 recorded the highest volume expansion ratio (2.6), while IR841 recorded the least (1.4) (Table 1). Nonparboiled samples recorded higher VER (2.28) than parboiled samples, but there was no steady decrease in the VER with steaming time although samples steamed at 45 min recorded the lowest VER (2.02) (Table 2). Nonparboiled NERICA1 recorded the highest VER (2.8), while IR841 steamed at 5 min recorded the lowest (1.2) (Table 5). VER was negatively correlated with lipid content (*R* = −.30, *p* < .001) and positivity correlated with AAC (*R* = .47, *p* < .0001), protein (*R* = .45, *p* < .0001), and sodium (*R* = .32, *p* < .001) contents (Table 4). Changes in the above cooking quality attributes with steaming time can be explained through an understanding of the gelatinization process (Bhattacharya, 1969; Derycke, Vandeputte, Vandeputte, et al., 2005) In nonparboiled rice, starch granules are separated by intergranular spaces which absorb water quickly during the cooking process. During cooking, the granules absorb water with a proportional swelling that ends with granule deformation and paste formation. In excess water, grains become soft upon complete gelatinization. In parboiled rice, some grain granules are partially gelatinized, sealing up the intergranular spaces and existing fissures on the grain (Bhattacharya, 1969; Ndindeng et al., 2014). This forms compact grains which absorb water less during cooking with a consequent decrease in the swelling rate and softening of the rice grains. Furthermore, amylose and protein barriers have also been suggested to restrict heat‐induced swelling during cooking or parboiling (Derycke, Vandeputte, Vandeputte, et al., 2005) explaining why parboiled samples recorded lower VER compared to the nonparboiled counterparts. Samples steamed for 35 min appeared to favor the absorption of more water that caused the starch to swell more than the other parboiled samples. Thirty‐five (35) min steaming time appeared to be that time when the starch granules undergo considerable changes, and this needs further investigation.

### 3.7 Total starch content (TSC), lipids, proteins, and minerals

#### 3.7.1 Total starch content (TSC)

NERICA7 recorded the lowest quantity of TSC (80%), while NERICA1 recorded the highest (88.7%) (Table 1). However, steaming time did not affect the TSC (Table 2) indicating that variety was the only factor affecting TSC in this study. NERICA1 steamed for 15 min recorded the highest TSC (92.6%), while NERICA7 steamed for 35 min recorded the least (77.3%) (Table 5). NERICA7 steamed for 35 min has been shown to record the lowest plasma glucose level 30 min after feeding in rats compared to other treatments (Zohoun et al., 2018). TSC correlated negatively with protein content (*R* = −.33, *p* < .001) and positively with AAC (*R* = .36, *p* < .001) (Table 4).

#### 3.7.2 Lipid content

WITA4 recorded the highest quantity of lipids (1.6%), while NERICA1 recorded the least (0.73%) (Table 1). Parboiling increased the lipid content of parboiled samples compared to nonparboiled counterparts, but no difference was observed among parboiled samples steamed at different steaming times when variety was not a factor (Table 2). WITA4 steamed for 35 min recorded the highest lipid content (1.86%), while nonparboiled IR841 recorded the least (0.25%) (Table 5). High quantities of free fatty acids in parboiled rice compared to nonparboiled have been documented (Nicolosi, Rogers, Ausman, & Orthoefer, 1993) although the amount of these free fatty acid will also depend on the variety and the parboiling steaming time as shown in the present study. Lipid was negatively correlated with protein (*R* = −.42, *p* < .0001) (Table 6), chalkiness, viscosities, cooking time, VER, phosphorus, sodium, and TSC and positively correlated with ASV and WUR. This suggests that a rice grain with high lipid content is more likely to suffer from breakages during milling, cook fast, soft, and hydrolyze faster.

**Table 6:**
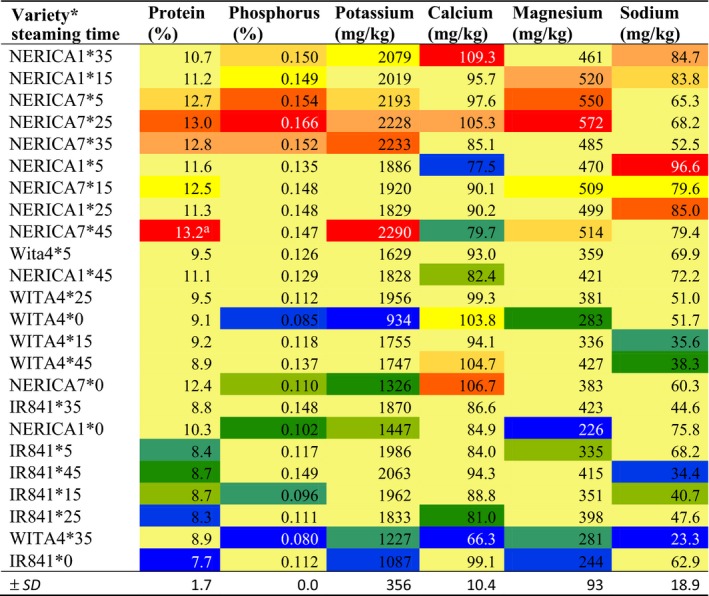
Interactive effect of variety and parboiling steaming time at atmospheric pressure on protein, phosphorus, potassium, calcium, magnesium, and sodium contents of some varieties cultivated in Benin

Values in red are highest, while those in blue are lowest.

#### 3.7.3 Protein content

NERICA7 recorded the highest protein content (12.8%), while WITA4 recorded the least (9.20%) (Table 1) Nonparboiled samples recorded lower protein content (9.91%) compared to parboiled samples (10.3%–10.6%) (Table 2), and the protein content was variety dependent. The highest protein content was recorded with NERICA7 steamed for 45 min (13.2%) and the lowest with nonparboiled IR841 (7.7%) (Table 6). Protein content was positively correlated with HRY, hardness, stickiness, cooking time, VER, phosphorus, potassium, magnesium, and sodium and negatively correlated with AAC, ASV, WUR, TSC, and lipids (Table 4). The higher protein content recorded for NERICA7 and NERICA1 confirms previous findings by Watanabe, Futakuchi, Jones, and Sobambo (2006) where higher protein content was recorded for upland NERICAs.

#### 3.7.4 Mineral content

Among varieties studied, NERICA7 recorded the highest quantity of phosphorus (0.146%), potassium (2031 mg/kg), and magnesium (502 mg/kg). NERICA1 recorded the highest quantity of sodium (83 mg/kg). WITA4 recorded the lowest quantity of phosphorus (0.110%), potassium (1541 mg/kg), magnesium (344 mg/kg), and sodium (45 mg/kg) (Table 1). Parboiled samples recorded higher quantities of phosphorus, potassium, magnesium, and sodium than nonparboiled samples, but no difference was observed among parboiled samples with varying parboiling steaming time (5 to 45 min) except for sodium (Table 2) that decreased with steaming time. Samples steamed for 35 and 45 min tended to record lower sodium content compared to sample steamed for 25 min or below. Samples steamed for 5 min recorded the highest sodium content (75 mg/kg), while those steamed for 35 min recorded the lowest (51.30 mg/kg).

NERICA7 steamed at 25 min recorded the highest phosphorus content (0.166%), while WITA4 steamed at 35 min recorded the lowest (0.08%) (Table 6). NERICA7 steamed at 45 min recorded the highest potassium content (2,290 mg/kg), while nonparboiled WITA4 recorded the lowest (934 mg/kg). NERICA7 steamed for 25 min recorded the highest magnesium content (572 mg/kg), while nonparboiled NERICA1 recorded the lowest (226 mg/kg). NERICA1 steamed for 5 min recorded the highest sodium content (96.6 mg/kg), while WITA4 steamed for 35 min recorded the lowest (23.3 mg/kg).

Studies have shown that, during parboiling, water‐soluble nutrients diffuse from the outer layer of the grain and from the husk into the endosperm explaining the high content of water‐soluble minerals in parboiled compared to nonparboiled samples. This is very important nutritionally as the loss of these nutrients during cooking in parboiled rice has been shown to be lower than in nonparboiled rice (Doesthale, Devara, Rao, & Belavady, 1979). In the present study, the diffusion of phosphorus, potassium, and magnesium occurred mainly during the soaking process as the amount of these minerals did not change with steaming time. Grain phosphorus, potassium, and magnesium correlated positively with each other and with HRY, hardness, cooking time, and proteins and negatively with chalkiness, viscosity, AAC, WUR, and lipids (Table 4). These results suggest that these three minerals may play important roles in starch structure during grain‐filling, parboiling, or cooking processes by forming specific linkages between starch (most probably amylopectin) and protein molecules (Derycke, Vandeputte, Vermeylen, et al. (2005); Derycke, Vandeputte, Vandeputte, et al. (2005)). This may explain why samples with high quantities of these minerals and proteins recorded higher HRY, cooking time, and cooked grain hardness. More studies are however needed to identify the specific proteins involved and how these proteins interact with starch and the metals quantified to influence the packing of starch molecules in chalky and nonchalky grains.

## 4 CONCLUSION

The effect of variety, steaming time, and the interaction of variety and steaming time were investigated on physicochemical and nutritional properties of two upland NERICA and two sativa rice varieties. Steaming time, variety, and interaction between variety and steaming time affected the physicochemical and nutritional properties of rice. Parboiled NERICA7 recorded the highest HRY when steaming was performed for 25 min. This variety may be suitable for processors looking to achieve high milling recoveries after parboiling. Nonparboiled IR841 recorded the shortest cooking time and may be suitable for consumers who want their rice to cook fast to save time. NERICA1 steamed for 45 min was the hardest, while nonparboiled IR841 was the softest. Nonparboiled NERICA7 was the stickiest, while NERICA1 steamed for 5 min was the least sticky. These treatments (varieties and processing options) provide a wide choice of hardness and stickiness products from which consumers can choose. NERICA7 demonstrated the highest PV, FV, and SBV values during the different steaming times. NERICA7 also demonstrated a high BV between 0 and 15 min steaming time but the lowest BV from 25 min of steaming. These results suggest that a proportion of the starch granules in NERICA7 remained ungelatinized (after steaming for 45 min at atmospheric pressure) probably due to a much stronger protein barrier. NERICA7 steamed for 35 min recorded the lowest TSC, and low TSC may be useful for people who want to maintain a low postprandial glucose level after rice consumption. NERICA7 steamed for 25 and 45 min recorded the highest phosphorus, magnesium, and potassium contents, respectively, and opens the possibility of using this variety and steaming times to reduce the effects of malnutrition due to deficiency in these minerals. The high amount of protein and minerals in parboiled NERICA7 makes this variety unique as it may well serve people with special needs. The results recorded in this study open a new area of screening other rice varieties for possible nutritional benefits using parboiling steaming time. Thus, depending on the desired physicochemical and nutritional property, specific varieties and parboiling treatments can be selected to achieve the outcome. Studies on effect of parboiling steaming time on in vivo digestibility of these varieties are ongoing and will be reported in due course. Furthermore, it will be interesting to investigate the mechanism behind the accumulation of proteins and minerals (phosphorus, potassium, and magnesium) in NERICA7 and how these affect chalkiness, grain breakage, and pasting behavior.

## CONFLICT OF INTEREST

None declared.
